# iNAP 2.0: Harnessing metabolic complementarity in microbial network analysis

**DOI:** 10.1002/imt2.235

**Published:** 2024-09-23

**Authors:** Xi Peng, Kai Feng, Xingsheng Yang, Qing He, Bo Zhao, Tong Li, Shang Wang, Ye Deng

**Affiliations:** ^1^ CAS Key Laboratory for Environmental Biotechnology, Research Center for Eco‐Environmental Sciences Chinese Academy of Sciences (CAS) Beijing China; ^2^ College of Resources and Environment University of Chinese Academy of Sciences Beijing China

**Keywords:** metabolic complementarity, metagenome‐assembled genomes, metabolic network

## Abstract

With the widespread adoption of metagenomic sequencing, new perspectives have emerged for studying microbial ecological networks, yielding metabolic evidence of interspecies interactions that traditional co‐occurrence networks cannot infer. This protocol introduces the integrated Network Analysis Pipeline 2.0 (iNAP 2.0), which features an innovative metabolic complementarity network for microbial studies from metagenomics sequencing data. iNAP 2.0 sets up a four‐module process for metabolic interaction analysis, namely: (I) Prepare genome‐scale metabolic models; (II) Infer pairwise interactions of genome‐scale metabolic models; (III) Construct metabolic interaction networks; and (IV) Analyze metabolic interaction networks. Starting from metagenome‐assembled or complete genomes, iNAP 2.0 offers a variety of methods to quantify the potential and trends of metabolic complementarity between models, including the PhyloMint pipeline based on phylogenetic distance‐adjusted metabolic complementarity, the SMETANA (species metabolic interaction analysis) approach based on cross‐feeding substrate exchange prediction, and metabolic distance calculation based on parsimonious flux balance analysis (pFBA). Notably, iNAP 2.0 integrates the random matrix theory (RMT) approach to find the suitable threshold for metabolic interaction network construction. Finally, the metabolic interaction networks can proceed to analysis using topological feature analysis such as hub node determination. In addition, a key feature of iNAP 2.0 is the identification of potentially transferable metabolites between species, presented as intermediate nodes that connect microbial nodes in the metabolic complementarity network. To illustrate these new features, we use a set of metagenome‐assembled genomes as an example to comprehensively document the usage of the tools. iNAP 2.0 is available at https://inap.denglab.org.cn for all users to register and use for free.

## Highlights

- The updated integrated Network Analysis Pipeline (iNAP 2.0) is an advanced platform that facilitates the calculation of various metabolic complementarity and competition indices, including the PhyloMint index, SMETANA (species metabolic interaction analysis), and metabolic distance, as well as the construction and analysis of metabolic interaction networks.
- Innovatively, iNAP 2.0 uses the random matrix theory (RMT) approach to determine the threshold for metabolic interaction networks.
- PhyloMint PTM in iNAP 2.0 allows users to view potentially transferable metabolites between microbial interactions and integrate them as network nodes to construct a microbe‐metabolite bipartite network alongside the microbial interaction network.

## Graphical abstract

The graphical abstract illustrates the advanced capabilities of iNAP 2.0, an integrated network analysis platform designed for comprehensive metabolic interaction studies. iNAP 2.0 enables the calculation of various metabolic indices, including the PhyloMint complementarity and competition index, SMETANA (species metabolic interaction analysis) scores, and metabolic distance, facilitating in‐depth analysis of metabolic complementarity and competition. It innovatively employs the random matrix theory (RMT) method to determine thresholds for constructing robust metabolic interaction networks. Additionally, the platform introduces PhyloMint PTM, which identifies potentially transferable metabolites between microbial interactions, integrating them into microbe‐metabolite bipartite networks alongside traditional microbial interaction networks. This combined approach provides a holistic view of microbial metabolic exchanges, making iNAP 2.0 a powerful tool for studying microbial ecology and inferring its metabolic keystones.

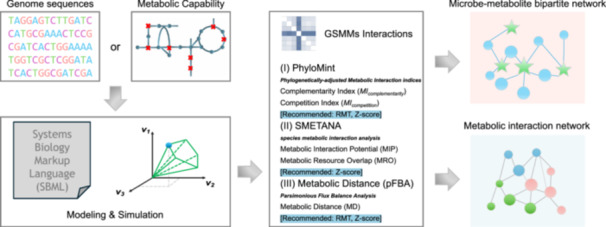

## INTRODUCTION

Microbial ecology research has benefited from advancements in sequencing techniques, ranging from marker gene‐based amplicon sequencing to shotgun metagenomics and sophisticated analysis methods, including taxonomic identification, diversity analysis, and microbial co‐occurrence networks [1]. While these methods, especially microbial ecological network analysis or microbial interaction networks, provide valuable insights, they often fall short of revealing the underlying mechanisms governing microbial interactions due to their reliance on statistical inference and limited capacity to capture functional details [2, 3]. However, with the rapid advancement in metagenomic data analysis, approaches based on functional traits or metabolic profiling now allow for a deeper understanding of microbial ecological networks. This progress has paved the way for new bioinformatic tools that enable the development of various modeling methods capable of predicting or simulating metabolite exchange or metabolic complementarity between microbes [4]. These methods rely on the information encoded within microbial genomes (e.g., reference genomes, metagenome‐assembled genomes, or single‐amplified genomes) and their genome‐scale metabolic models (GSMMs) [5]. The level of detail used varies, with some methods focusing solely on the types of reactions and metabolites, while others consider factors like microbial growth rates. Specifically, PhyloMint focuses on the types of metabolites in pairwise genomes, SMETANA (species metabolic interaction analysis) focuses on the overlap and exchange of metabolic resources in communities (can be more than two species, higher order interactions), and metabolic distances are calculated by simulating metabolic fluxes in the metabolic models on growth or energy production.

**Figure 1:**
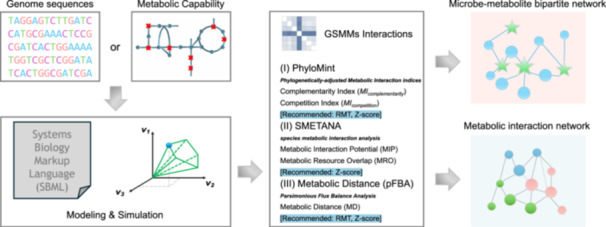
An overview of the schematic design and implemented tools of metabolic interaction network analysis of iNAP 2.0. Users can start from genome or protein sequence to reconstruct genome‐scale metabolic models and proceed to multiple analyses. iNAP, integrated Network Analysis Pipeline.

Although these bioinformatic tools can construct and analyze GSMMs, they generally require specialized software or programming languages such as Python, MATLAB, or command‐line interfaces. This poses a significant barrier for researchers without expertise in these areas, hindering large‐scale and specialized data analysis processes. In addition, there were no critical methods or criteria to detect the microbial interactions characterized by the massive amount of modeling‐derived data (primarily numerical), where the random matrix theory (RMT) approach can effectively address the need. To meet these challenges, we integrated several user‐friendly tools into the well‐established integrated Network Analysis Pipeline (iNAP) [6], now upgraded to iNAP 2.0. These tools include CarveMe for automated GSMM construction [7], PhyloMint [8], SMETANA [5], and Cobrapy [9] for predicting metabolic exchanges between microbes (Figure 1). To put it in a nutshell, the iNAP 2.0 update offers significant improvements over its predecessor, equipping genome‐scale metabolic modeling and metabolic interaction as new weapons to decipher microbial ecological networks of statistical correlations.

### Workflow overview and implementation

**Figure 2:**
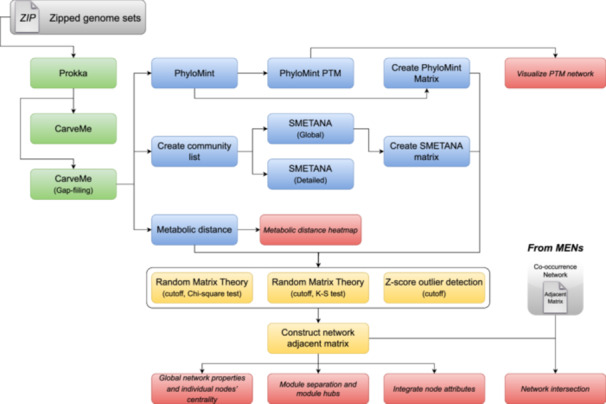
The workflow of metabolic modeling analysis and construction of complementarity/competition metabolic network. PTM, potentially transferable metabolite; K‐S test, Kolmogorov–Smirnov test; MENs, molecular ecological networks.

The metabolic modeling pipeline added in iNAP 2.0 allows users to start the analysis from genome sequences (Box 1, Figure 2). The pipeline leverages Prokka for predicting coding sequences [11] and CarveMe for the automated construction of GSMMs. Alternatively, users can manually curate and reconstruct GSMMs using tools like ModelSEED [12], or directly import prebuilt models from databases like the Virtual Metabolic Human (VMH) [13]. For analysis of metabolic interactions between microbes, iNAP 2.0 offers three methods: PhyloMint (competition/complementarity index), SMETANA score, and metabolic distance based on pFBA. Notably, the PhyloMint index highlights potentially transferable metabolites considered during calculations [8]. Different from the arbitrary threshold determination for the metabolic interaction index, iNAP 2.0 provides the RMT method, which could find a fair threshold of statistical significance, to construct and analyze the topological properties of the resulting metabolic interaction network.

Box 1: **Data set formats for input files**

This box gives the input file format required for genome‐based metabolic modeling analysis.

Zipped genome sets (.zip). The zipped genome set contains all genome sequence files (.fasta/.fa) to be analyzed. Ensure all sequence files are directly compressed rather than stored in folders and then compressed. Each sequence file name should be unique, not starting with numbers, and not contain spaces, hyphens, or other special characters (underscore is recommended). If genome sets are planned for SMETANA analysis, the number of genome files should not exceed 300 due to SMETANA's high consumption of computational resources.

Prokka predicted protein sequences (.zip). The predicted protein set contains all protein sequence files (.faa) corresponding to the genomes. This file can be obtained using the Prokka tool or protein sequence files already obtained or downloaded from reference databases. Compression and naming requirements are the same as above.

Growth medium for gap‐filling (.txt/.tabular). When using CarveMe (gap filling) in Step 2‐B, users can upload customized media in addition to the default five media. The media description file should contain four columns: medium, description, compound, and name. Note that compound names and IDs must be consistent with the BiGG database (http://bigg.ucsd.edu/, [10]).

Inheriting the user‐friendly Galaxy framework [14] from its predecessor, iNAP 2.0 provides a detailed protocol outlining the analysis steps. This protocol describes the method parameters selection and results of each modeling method in an easy‐to‐follow order, along with potential avenues for further exploration.

#### Section I: Prepare GSMMs

According to the requirements for the input file format (Box 1), users need to upload the zipped genome sets and start from Step 1, or upload the zipped protein sequence sets and start from Step 2‐A/B. For a better startup, two zipped demo genome sets are stored in: User Panel—Shared Data—Data Libraries—Metabolic Modeling Demo Datasets. Demo data set 1 is a seven‐strain microbiome genome set (a SIHUMI, simplified human microbiome) [15]. The reference genomes were downloaded from the NCBI Reference Sequence Database (RefSeq). Demo data set 2 is a genome set of 100 metagenome‐assembled genomes (MAGs) from a hot spring habitat [16].

1. *Prokka*. iNAP 2.0 utilizes Prokka with default settings for genome annotation [11]. Alternatively, users can employ tools like Prodigal [17] or EGGNOG‐mapper [18, 19] for this step. The output is a compressed protein sequence file (Box 1).

2‐A. *CarveMe*. iNAP 2.0 offers CarveMe, a fast and automated tool for building GSMMs [7]. This step takes the zipped protein sequence file output from Step 1 or zipped predownloaded annotated protein sets as input. The output format (sbml‐fbc2) ensures compatibility with most constraint‐based modeling tools. In the GSMM reconstructed by CarveMe, the upper and lower bounds of the reaction flux (ready for FBA) directly call the default values in Cobra (*cobra_default_ub, cobra_default_lb*), which are 1000 and −1000 mmol/gDW/h. The output file of this step is a zipped metabolic model file (XML format).

2‐B. *CarveMe (gap filling)*. When CarveMe is used to construct a GSMM, the program determines the scores of various reactions based on the functional profiles. It performs mixed integer linear programming (MILP) to determine which reactions are supposed to be included in the final model. Therefore, microbial models in natural environments might lack certain reactions due to limitations in binning or annotation (Box 2). We recommend using the gap‐filling function to correct the model derived from metagenome‐assembled genomes (MAGs) of environmental metagenomes. CarveMe provides five predefined media compositions for gap filling (default: Lysogeny broth, LB), allowing growth simulation of the model on corresponding media. Users can also create and utilize custom media compositions, such as dietary components for gut microbiome models. It is important to note that defining media for environmental microbiomes can be challenging due to the difficulty of culturing most microorganisms and the limitations of rich media. Using rich media for gap‐filling can ease over‐gap‐filling. We also recommend using some tools, for example, CHESHIRE (CHEbyshev Spectral HyperlInk pREdictor), based on deep learning methods to assist gap filling for refining metabolic models [20]. The input and output files are the same as in Step 2‐A. Users are suggested to declare the media composition used for gap‐filling in their results to guarantee the reproducibility of the modeling.

Box 2: **Note on GSMM reconstruction quality**

While GSMM reconstruction is a powerful tool, it is not without its limitations. Several factors can influence the model quality, and it is important to acknowledge that our pipeline does not support GSMM reconstruction quality evaluation. However, the credibility of GSMMs can be enhanced through the following strategies:

1. Utilizing high‐quality genomes: The reliability of a GSMM is closely tied to the quality of the genomes used. Genomes vary in quality, from complete reference genomes to MAGs. Whenever possible, whole reference genomes should be prioritized for GSMM reconstruction due to their completeness. However, given that many microbes cannot be isolated and cultured under laboratory conditions, MAGs serve as a valuable alternative. Recent studies, such as those by Giordano et al. [21] and Hsieh et al. [22], have demonstrated the successful use of MAGs in GSMM reconstruction, underscoring their credibility. Nevertheless, it is crucial to emphasize that the quality of MAGs, particularly their completeness, significantly impacts modeling accuracy. Therefore, stricter criteria should be applied when refining MAGs for GSMM reconstruction.
2. Choosing reliable model builders: The choice of GSMM reconstruction tools can also affect model quality. Mendoza et al. conducted a systematic assessment of GSMM tools, offering users four key criteria: findability, accessibility, interoperability, and reusability [23]. Based on these criteria, tools like CarveMe were highlighted for their ability to generate GSMMs with high reaction set similarity to manually curated models. In this protocol, we encourage users to explore various model builders. However, researchers should remain vigilant about the potential for false results, particularly when interpreting outcomes at the gene level.

#### Section II: Infer pairwise interactions of GSMMs

iNAP 2.0 offers three methods to quantify metabolic interactions between pairwise GSMMs: PhyloMint, SMETANA, and pFBA‐based metabolic distance. Like species abundance‐based co‐occurrence networks, these metabolic interaction indices can be filtered and constructed as microbial metabolic networks. This section explains the principles and interpretations of each method.

##### Method 1: PhyloMint

PhyloMint predicts metabolic indices of competition and complementarity (*MI*_competition_ and *MI*_complementarity_) between genomes using their GSMMs. Each GSMM first predicts a seed set of metabolites, the minimal subset of compounds that cannot be synthesized endogenously (defined as a strongly connected component, SCC). Then, the competition and complementarity indices between pairwise models A and B are calculated using the formula given in [8]:

$${\mathrm{MI}}_{\mathrm{competition}(A,B)}=\frac{\sum C\left({\mathrm{SeedSet}}_{A}\cap {\mathrm{SeedSet}}_{B}\right)}{\sum C\left({\mathrm{SeedSet}}_{A}\right)},$$

$${\mathrm{MI}}_{\mathrm{complementarity}(A,B)}=\frac{\left|{\mathrm{SeedSet}}_{A}\cap {\neg \mathrm{SeedSet}}_{B}\right|}{\left|{\mathrm{SeedSet}}_{A}\cap ({\mathrm{SeedSet}}_{B}\cup {\neg \mathrm{SeedSet}}_{B})\right|},$$

where *C* is the inverse of the seed set size, and ¬SeedSet_*B*_ is the nonseed set of B. According to the calculation formula, *MI*_competition_ and *MI*_complementarity_ are asymmetric.

3. *PhyloMint*. The program calculates *MI*_complementarity_/*MI*_competition_ between *n* input GSMMs (output from Step 2‐A/B), resulting in *n* ^2^ sets of indices calculated. These indices show how many models A and B share in metabolic functions and how well A can use substances released by B (Table 1). The parameter *MaxCC* indicates the maximum number of members in an SCC (recommended set to default: 5). The result of PhyloMint is stored in a four‐column table, showing genome pairs and their *MI*_competition_ and *MI*_complementarity_.

**Table 1:** Brief introduction of three approaches of metabolic interaction modeling and their representing index explanation.

Method	Index	Meaning	Range	Symmetry	Reference
PhyloMint	*MI*_competition_	The baseline metabolic overlap between two GSMMs.	[0,1]	Asymmetric	[8]
	*MI*_complementarity_	The potential for one GSMM to utilize the other's potential metabolite output.	[0,1]	Asymmetric	
SMETANA	MIP (Metabolic interaction potential)	The difference in minimal nutritional requirements when a community allows metabolite exchange and when it does not at all.	Positive integers	Symmetric	[5, 24]
	MRO (Metabolic resource overlap)	The maximum possible overlap between the minimal nutritional requirements of all member species.	[0,1]	Symmetric	
	SCS (Species coupling score)	The dependency of one species in the presence of the others to survive.	[0,1]	Symmetric	
	MUS (Metabolite uptake score)	The dependency of one species in the specific metabolite given by the others.	[0,1]	Symmetric	
	MPS (Metabolite production score)	The ability of one species to produce a specific metabolite.	Binary	Symmetric	
	SMETANA score	The certainty on a cross‐feeding interaction.	[0,1]	Symmetric	
Metabolic distance	*MD*	The dissimilarity between vectors of reaction fluxes of two GSMMs.	[0, ∞)	Symmetric	[25]

Abbreviation: GSMM, genome‐scale metabolic model.

4. *PhyloMint PTM*. *MI*_complementarity_ index, as noted earlier, represents A's potential to utilize metabolic substances from B. Potentially transferable metabolites (PTMs) are defined as the intersection of A's seed set and B's nonseed set, as per [8]. This step takes in the zipped model set (output of Step 2‐A/B) and PhyloMint result (output of Step 3) as inputs and outputs these substances in a tabular file, displaying the donor and receptor of each compound along with their nomenclature and full index in the BiGG database. Note that if your GSMMs are obtained elsewhere instead of generated by CarveMe, the file extension might be in SBML format (Systems Biology Markup Language). Remember to change the extension setting in this step.

5. *Create PhyloMint Matrix*. This step converts the result generated by Step 4 from a tabular form to a matrix form for network threshold determination. In particular, we provide two processing modes for the asymmetric *MI* index: (1) Keep the original value, that is, directly convert the results of Step 4 into an asymmetric matrix, and then the metabolic interaction network constructed using this matrix will be directed; (2) Select the larger value in the pairwise index of each pair of models to represent the interaction strength between the models, so that the generated matrix is symmetric, and the metabolic interaction network constructed using it will be undirected, which we set to default and recommend for better using the RMT‐based method for network threshold determination.

##### Method 2: SMETANA

SMETANA is a tool to quantify competition and complementation within communities by calculating metabolic resource overlap and minimum growth requirement metabolites in the community [5]. SMETANA allows the simulation of communities composed of more than or equal to two GSMMs, so the program (Step 7 and 8) requires a table to indicate which GSMMs belong to a community. On the iNAP 2.0 platform, the limit of models for SMETANA analysis is 300.

6. *Create Community List*. To calculate the interaction between pairwise GSMMs, iNAP 2.0 provides the required table of all pairwise GSMMs in a given model set (output of Step 2‐A/B) by default, which will be used as input for Steps 7(‐A/B) and 8(‐A/B).

7‐A. *SMETANA Global*. For a given community, SMETANA defines two indices, metabolic resource overlap (MRO) and metabolic interaction potential (MIP), to represent the competition and complementarity levels of the community, respectively. The specific definition and calculation of the indices have been previously described in detail [5]. The indices can be calculated using the global mode of SMETANA. Users should input the zipped genome sets and the community list obtained in Step 6 to generate the MIP/MRO values of the pairwise models in the community.

7‐B. *Iterative SMETANA Global*. SMETANA has been confirmed by its developers to have a drawback: the results of each run may vary slightly. This inconsistency is due to the solution pool feature of the CPLEX solver. [Correction added on 27 September 2024, after first online publication: In the preceding sentence, the word “consistency” was changed to “inconsistency”.] The developers recommend running multiple runs and calculating the average index value to represent the final value. To achieve this, the program is designed to run 2‐10 runs of the same input as Step 7‐A and output the average results.

8‐A. *SMETANA Detailed*. In addition to using MRO/MIP to quantify the metabolic interactions of the community, SMETANA also provides a detailed mode to calculate a series of indices to quantify further the interspecies interactions: SCS (species coupling score), metabolite uptake score (MUS), and metabolite production score (MPS). These three indices are combined and recorded as the SMETANA score to represent the sum of interspecies dependencies in the community. The definitions and meanings of all indices are detailed in Zelezniak et al. [5] and Table 1. The input request is consistent with Step 7‐A.

8‐B. *Iterative SMETANA Detailed*. The reason for iterative calculation is the same as Step 7‐B.

9. *Create SMETANA Matrix*. This step converts the SMETANA MIP/MRO results from a tabular to two matrices for network threshold determination. It requires the community list (output from Step 6) and the SMETANA global mode results (output from Step 7‐A/B) as input files.

##### Method 3: Metabolic distance

Previous studies have suggested that metabolic distance (metabolic dissimilarity) is vital in forming and determining synergistic interactions in microbial communities [25, 26].

10. *Metabolic distance*. This program helps to calculate the pairwise metabolic distances of GSMMs according to the method described by Giri et al. [25]. The input for this step is the zipped model sets (output of Step 2‐A/B). Specifically, the program first conducts FBA on each model with the biomass reaction as the objective function to optimize (maximize) the biomass reaction flux, which is typically used to represent the growth rate reaction. Optimizing other objectives, such as ATP yield, could lead to different metabolic strategies. By default, iNAP 2.0 fills in “Growth”, representing the growth rate reaction in GSMMs generated by CarveMe. Then, the optimized biomass reaction flux is fixed, and a parsimonious FBA (pFBA) is conducted to minimize the sum of absolute fluxes in each model while constraining the objective function (e.g., biomass production) to the optimal value obtained from the initial FBA. Subsequently, the reactions whose flux is not zero in at least one model are selected as representatives, the flux vectors of the models are generated, and the Euclidean distances between them are calculated as proxies of metabolic distances. This step outputs the results in three forms: two distance matrixes (original Euclidean distance and standardized Euclidean distance) and a three‐column table of the original Euclidean distance (model A/model B/Euclidean distance). One should inspect the original Euclidean distance first. If the matrix contains many values that differ by order of magnitude (this is likely to happen when models built with different bounds are input simultaneously, which we do not recommend), using the standardized Euclidean distance matrix should be considered. The standardized Euclidean distance is defined as the Euclidean distance calculated on the standardized data. The standard data is calculated according to this formula: Standard value = (Original value – Mean value)/Standard deviation [27].

#### Section III: Construct metabolic interaction networks

Different metabolic interaction indices have different numerical forms and value ranges. iNAP 2.0 provides various methods to determine network thresholds, including RMT‐based approaches.

11. *RMT (cutoff, Chi‐square test)*. This step can use the adjacency matrix representing the interaction strength generated by PhyloMint (Step 5) and metabolic distance (Step 10) as input. Since the values of SMETANA indices do not meet the requirements of the RMT method, it is recommended to use the Z‐score method (See Step 13). Currently, this step only accepts symmetric matrices as input, and the values in the matrix should be normalized between 0 and 1.

12. *RMT (cutoff, Kolmogorov–Smirnov test)*. This step and Step 11 use the RMT‐based method to determine the network threshold. The *χ* ^2^ test is utilized in Step 11, and the Kolmogorov–Smirnov test is used in this step. Compared with the *χ* ^2^ test, the Kolmogorov–Smirnov test is expected to give a more relaxed threshold, which may be more practical when dealing with values like the *MI*_competition_ and *MI*_complementarity_ indices.

13. *Z‐score outlier detection*. iNAP 2.0 provides a Z‐score outlier detection method for the adjacent matrix to filter interactions for constructing the networks. This method has been used to build a network based on the PhyloMint *MI*_competition_ and *MI*_complementarity_ indices [8]. However, we recommend using this method for SMETANA indices (e.g., MIP) and metabolic distance (before normalization) because they might not fit the requirement of the RMT approach. The input for this step is the adjacency matrix representing the interaction strength generated by PhyloMint (Step 5), SMETANA Global (Step 7‐A/B), and metabolic distance (Step 10). We provide standard and modified Z‐score formulas for outlier detection:

$$Z_{\mathrm{standard}}=\frac{\left(x_{obs}-\mu \right)}{\sigma },$$

$$Z_{\mathrm{modified}}=\frac{0.6745\left(x_{\mathrm{obs}}-\tilde{x}\right)}{\mathrm{MAD}},$$

where *x*_obs_ is the observed value, *µ*, *σ*, $\tilde{x}$ and MAD are the mean, standard deviation, median and median absolute deviation of the data set. There are different reports on the filtering criteria for outliers: the absolute values of Z‐score greater than 3.5 [28] and 2.698 [29] are more commonly used.

14. *Construct Network Adjacent Matrix*. After selecting an appropriate method to determine the threshold for network filtering (selected by Steps 11, 12, or 13), the original adjacent matrix and the selected threshold are input into this step, which generates the adjacent matrix representing the final metabolic interaction network.

#### Section IV: Analyze metabolic interaction networks

All the tools that have been introduced in iNAP can be used for the analysis of metabolic interaction networks, such as *global network properties, individual nodes’ centrality*, *module separation, and module hubs*. Below, some new tools or analysis tools that are specific to metabolic interaction networks are introduced in detail.

**Figure 3:**
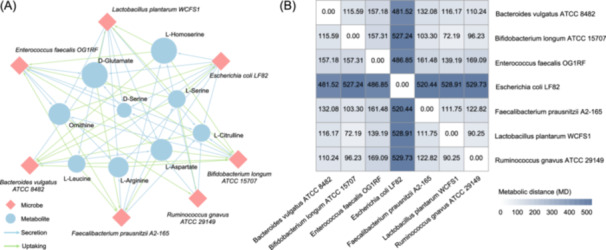
Visualization of the bipartite network generated by PhyloMint PTM and metabolic distance heatmap. (A) Microbe‐metabolite (potentially transferable metabolite) bipartite network output by iNAP 2.0. (B) A heatmap of metabolic distance generated by iNAP 2.0. The results were obtained from demo data set 1 [15]. iNAP, integrated Network Analysis Pipeline; PTM, potentially transferable metabolite.

15. *Global network properties and individual nodes’ centrality*. This step requires the adjacent matrix of metabolic interactions from Step 14 as input. Two result tabular files are (1) A global property file including many parameters such as average node degree, average clustering coefficient, and network density, which fully describe the properties of the metabolic interaction network; (2) A node attribute file including the properties of all nodes, such as degree, betweenness, stress, and so on.

16. *Module separation and module hubs*. This step also requires the adjacent matrix of metabolic interactions from Step 14 as input. It calculates the network modularity and distributes the nodes to different modules. Based on the module, each node's within‐module connectivity (*z*_*i*_) and among‐module connectivity (*P*_*i*_) will be computed. Based on the *z*_*i*_‐*P*_*i*_ distribution, the keystone nodes or hub nodes can be assigned, such as module hubs, connectors, and network hubs. Details can be found on the help page of this tool.

17. *Integrate node attributes*. The node centrality (output of Step 15), the node within‐ and among‐module connectivity (*z*_*i*_‐*P*_*i*_ value and roles in the network, output of Step 16), and the taxonomic annotation of the node (optional and uploaded by users, tabular‐separated. txt file with first column as node IDs, referred to example file in iNAP 2.0) are essential components of the microbial network information. This step can merge the above three files for subsequent analysis or as an annotation file for visualization.

18. *Network intersection*. This step compares the adjacent matrices of two networks and finds the subnetworks composed of their common edges. It then outputs the intersected network with an adjacent matrix and edge list of the subnetwork.

19. *Visualize the PTM network*. This step uses the output of Step 5 as input to transform the potentially transferable metabolites in the specific network into a directed bipartite microbe‐metabolite network (Figure 3A). All metabolite annotations stored in the input are integrated and output in a node attribute table.

20. *Metabolic distance heatmap*. This step generates a heatmap using the metabolic distance matrix (output of Step 10), which allows for the significant identification of distinctive metabolic profiles in microbial consortiums (Figure 3B).

#### Troubleshooting

The advice for troubleshooting is summarized in Table 2.

**Table 2:** Troubleshooting table. [Correction added on 27 September 2024, after first online publication: In the third row of “Error message/Problem encountered” column, the number “500” was revised to “300”.]

Step	Error message/Problem encountered	Possible reason	Solutions
All	An error occurred while updating information with the server.	The server is overloaded or under maintenance.	Wait patiently.
1, 2, 3	Error opening file: No such file or directory.	1. Wrong extension; 2. The input compressed file contains a folder.	1. Correct the extension; 2. Make sure all sequence files are directly zipped.
1, 2, 3	Error: Sequence file number exceeds the limit of 300.	Metabolic modeling may consume a lot of computing resources, and iNAP2.0 allows processing up to 300 genomes/models.	Reduce the number of genomes or consider using other computing resources such as HPC.
8, 9, 10, 11	The program takes forever.	SMETANA is a very time‐consuming program.	Reduce the number of model pairs.

Abbreviation: SMETANA, species metabolic interaction analysis.

## AUTHOR CONTRIBUTIONS

All authors contributed to the pipeline development and analysis workflow. The initial idea and framework were conceived by Xi Peng and Ye Deng. The upgraded tools were contributed to and maintained by Xi Peng and Kai Feng. During the pipeline development, Xingsheng Yang, Qing He, Bo Zhao, Tong Li, and Shang Wang contributed to the test phase. This protocol was written by Xi Peng and Kai Feng and revised by Ye Deng. All authors have read the final manuscript and approved it for publication.

## CONFLICT OF INTEREST STATEMENT

The authors declare no conflict of interest.

## ETHICS STATEMENT

No animals or humans were involved in this study.

## Data Availability

iNAP 2.0 is publicly accessible to all researchers (https://inap.denglab.org.cn) with open registration. Demo datasets for testing are stored in Shared Data. Supplementary materials (figures, tables, scripts, graphical abstract, slides, videos, Chinese translated version, and updated materials) may be found in the online DOI or iMeta Science http://www.imeta.science/.
